# T Helper 2-Associated Immunity in the Pathogenesis of Systemic Lupus Erythematosus

**DOI:** 10.3389/fimmu.2022.866549

**Published:** 2022-04-04

**Authors:** Haeun Ko, Chan Johng Kim, Sin-Hyeog Im

**Affiliations:** ^1^ Department of Life Sciences, Pohang University of Science and Technology, Pohang, South Korea; ^2^ Pohang University of Science and Technology (POSTECH) Biotech Center, Pohang University of Science and Technology, Pohang, South Korea; ^3^ Institute for Convergence Research and Education, Yonsei University, Seoul, South Korea; ^4^ ImmunoBiome Inc., Bio Open Innovation Center, Pohang, South Korea

**Keywords:** autoimmunity, SLE, lupus nephritis, Th2, IL-4, IgE, autoantibody

## Abstract

Systemic Lupus Erythematosus (SLE) is a chronic autoimmune disease that mainly affects women in their reproductive years. A complex interaction of environmental and genetic factors leads to the disruption of immune tolerance towards self, causing overt immune activation and production of autoantibodies that attack multiple organs. Kidney damage, termed lupus nephritis, is the leading cause of SLE-related morbidity and mortality. Autoantibodies are central to propagating lupus nephritis through forming immune complexes and triggering complements. Immunoglobulin G (IgG) potently activates complement; therefore, autoantibodies were mainly considered to be of the IgG isotype. However, studies revealed that over 50% of patients produce autoantibodies of the IgE isotype. IgE autoantibodies actively participate in disease pathogenesis as omalizumab treatment, a humanized anti-IgE monoclonal antibody, improved disease severity in an SLE clinical trial. IgE is a hallmark of T helper 2-associated immunity. Thus, T helper 2-associated immunity seems to play a pathogenic role in a subset of SLE patients. This review summarizes human and animal studies that illustrate type 2 immune responses involved during the pathology of SLE.

## Introduction

Systemic Lupus Erythematosus (SLE) is an autoimmune disease that affects multiple organs such as the skin, joints, kidney, heart, and brain (1). SLE mainly affects women in their reproductive years and shows higher prevalence in African, Hispanic, or Asian ethnicities. SLE prognosis has seen significant improvement in the last half-century, with survival probability reaching 92% during a 10-year period (2). However, patients with renal disease, termed lupus nephritis, have a poorer prognosis than those without renal complications (3). Thus, a large proportion of research is focused on understanding the mechanisms surrounding lupus nephritis. Autoantibodies are the primary cause for renal damage *via* induction of inflammation in the kidneys through immune complex deposition and complement activation (4). Indeed, autoantibodies specific for double-stranded DNA (anti-dsDNA) show a positive correlation with disease severity (5). Especially, autoantibodies of the IgG1 and IgG3 isotypes are considered pathogenic due to their superior ability to activate complement and engage Fc receptors (6). More than 50% of SLE patients, however, also produce autoantibodies of the IgE subclass, and these antibodies showed a strong association with disease severity (7). Moreover, treatment of SLE patients with monoclonal antibodies specific for IgE (omalizumab) improved disease activity in a randomized clinical trial (8). Thus, it seems IgE is also involved in the pathogenesis of lupus nephritis.

IgE is a type 2-associated immunoglobulin typically associated with allergic disease. Therefore, most IgE and type 2 immunity-associated responses have been described in the context of allergic disease. Briefly, exposure to allergens causes epithelial cells to produce alarmins (9). Alarmins activate type 2 innate lymphoid cells (ILC2) and dendritic cells (DCs) to produce type 2 cytokines and to induce T cell differentiation, respectively. Activated DCs induce the differentiation of T helper 2 (Th2) cells and T follicular helper (Tfh) cells. Th2 cells recruit innate immune cells such as eosinophils and mast cells to allergic sites, whereas Tfh cells activate B cells to produce IgE. Subsequently, IgE binds to allergens located in allergic sites, which are then recognized by innate immune cells *via* their IgE receptor, FcϵRI. This interaction activates innate cells to produce effector molecules that propagate inflammation (10).

**Figure 1:**
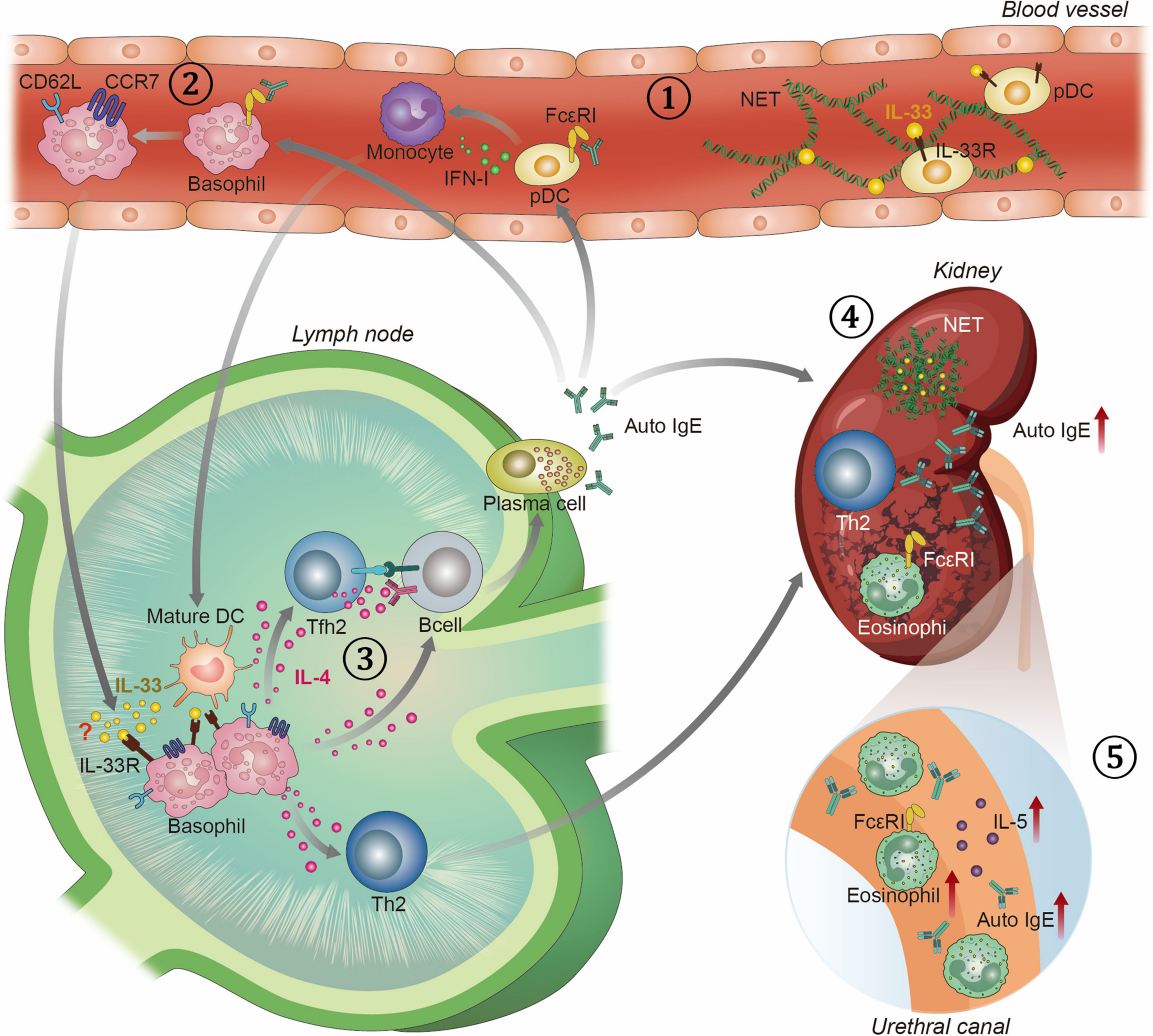
Overview of T helper 2 associated immune response in SLE. 1) SLE patients have elevated levels of NETs decorated with bioactive IL-33 and auto-IgE in blood. They are potent in activating pDCs to produce IFN-I, which are known to play key role in the pathogenesis of SLE. Activated pDCs stimulate monocytes to differentiate into mature DCs. 2) Auto-IgE also stimulates basophils to drain into SLOs by upregulating CD62L and CCR7. 3) In SLO, basophils polarize T cells into Th2 and Tfh2 cells, and activate B cells in IL-4 dependent manner. Tfh2 cells promote the differentiation of B cells into IgE autoantibody-producing plasma cells. IgE autoantibodies, in turn, activate pDCs, basophils, and eosinophils. 4) Once activated, Th2 cells and eosinophils infiltrate into the kidney and augment lupus nephritis. In the kidney, NETs decorated with IL-33 and auto-IgE are also found. 5) Eosinophils, auto-IgE, and IL-5 cytokines are also detected in the urine of SLE patients.

The type 2-associated immune response found in SLE shows similarities and differences with the response seen in allergic disease. This review discusses the type 2-associated immune response found in SLE patients and describes how this immune response is shaped during the induction phase and damages end organs during the effector phase (**Figure 1**).

## Induction Phase

### Alarmins

Alarmins act as the first line of defense in our body’s outer and inner extremities. They are constitutively expressed by structural and immune cells and are rapidly released upon sensing environmental triggers, such as viral infections, tobacco smoke, pollutants, and physical damage (11). Thus, alarmins play crucial roles in shaping the initial response of our immune system. However, excessive release of alarmins is detrimental to our health and can contribute to various pathologies such as tumorigenesis, allergy, and autoimmunity (11). For example, exposure to allergens or viral infections in the lung causes a rapid release of alarmins by epithelial cells, leading to the development of asthma. Asthma is a type 2-mediated inflammatory disease, and the alarmins interleukin-25 (IL-25) and interleukin-33 (IL-33) play essential roles in shaping the Th2 response signature found in asthma.

### Interleukin-25

IL-25 is produced by both epithelial cells and immune cells and is involved in the recruitment and activation of Th2 cells, ILC2, B cells, eosinophils, and mast cells. The IL-25 receptor is expressed on multiple immune cells and structural cells, such as endothelial cells, epithelial cells, and fibroblasts. Thus, once released, IL-25 acts in an autocrine feed-forward mechanism (12). Currently, there are two reports on the role of IL-25 in association with SLE (**Tables 1** and **2**). Both reports claim IL-25 expression is increased in the serum of SLE patients, especially in patients with active disease severity and lupus nephritis (13, 14). Moreover, serum IL-25 levels showed a positive correlation with disease severity and anti-dsDNA autoantibodies, suggesting IL-25 might play a pathogenic role in SLE patients (13, 14). However, the second report claims IL-25 plays a protective role in SLE as injection of recombinant IL-25 ameliorated symptoms in MRL/Lpr mice, while the addition of recombinant IL-25 to SLE PBMC cultures suppressed secretion of inflammatory cytokines (13). Moreover, serum levels of IL-25 had a negative correlation with serum levels of IL-5 and IgE in SLE patients (14). Thus, the current evidence suggests IL-25 is highly induced during SLE pathogenesis; however, IL-25 seems to play an immunosuppressive role and is not involved in inducing type 2 responses in SLE patients.

**Table 1:** Type 2-associated immune response in SLE patients.

Factor	Role in pathogenesis of SLE (Human)	Reference(s)
IL-25 (Alarmins)	- Increased serum IL-25 protein levels in SLE patients - Serum IL-25 had positive correlation with disease severity and anti-dsDNA autoantibodies in SLE patients - Recombinant IL-25 suppressed inflammtory cytokine secretion from SLE PBMC cultures - Serum IL-25 cytokine had negative correlation with serum IL-5 and IgE levels in SLE patients	(13, 14)
IL-33 (Alarmins)	- Increase of neutrophil extracellular traps (NETs) decorated with IL-33 in blood of SLE patients - NETs with IL-33 showed positive correlation with SLEDAI scores - NETs with IL-33 were detected in inflamed skin and inflamed kidney of SLE patients	(15)
Basophil	- Basophil counts were reduced in circulation and accumulated in lymphnodes and spleen of SLE patients - Basophils are potential primary source of IL-4 in secondary lymphoid organ - Basophils isolated form SLE patients induced anti-dsDNA IgG and anti-dsDNA IgE *in vitro*	(16, 18)
Th2 cells	- Frequency of Th2 cells were increased in blood of SLE patients - Th2 cells were detected in kidney biopsies of Lupus nephritis patients - IL-4 levels positively corrlated with hypercellullarity in nephritis patients	(16, 19, 20)
Tfh cells	- The number of Tfh and Th17 cells positively correlated with disease activity in SLE patients - Low dose of rhIL-2 administration ameliorated disease activity in patients by increasing the number of Tregs and decreasing the number of Tfh and Th17 cells	(21–24)
Tfh2 cells	- The frequencies of Tfh2 cells were significantly higher in active lupus patients than healthy control - The number of Tfh2 cells positively correlated with SLEDAI scores in SLE patients - Tfh2 cells were highly activated in SLE patients and were better at inducing plasma cell differentiaiton in vitro than control group	(25, 26)
Anti-dsDNA IgE	- 50-60% of cohort were positive for autoreactive IgE, and antibody titers correlated with SLEDAI scores and active nephritis - 35% of lupus nephritis patients showed IgE deposition in kidney and had poorer prognosis. - Patients with IgE deposition showed upto 71% increase in occurence rate of lupus nephritis	(7, 27)
Eosinophil	- Increase in urinary eosinophils and IL-5 in lupus nephritis patients. - Eosinophil level showed correlation with renal function and SLE disease activity. - Patients with eosinophiluria and tissue eosinophil infiltration were more likely to progress to end-stage kidney disease. - Blood eosinophil levels between SLE patients and healthy controls were comparable.	(28, 29)

**Table 2:** Type 2-associated immune response in SLE mouse model.

Factor	Role in pathogenesis of SLE (Mouse)	Reference(s)
IL-25 (Alarmins)	- Injection of recombinant IL-25 ameliorated lupus symptoms in MRL/Lpr mice	(13)
IL-33 (Alarmins)	- Treatment with IL-33 inhibitory antibodies alleviated lupus symptoms in MRL/Lpr mice - Early tratment of recombinant IL-33 alleviated SLE symptoms in NZB/W F1 mice	(30, 31)
Basophil	- Activated basophils and IgE autoantibodies play role in autoantibody production and lupus nephirtis development in *Lyn-/-* mice - IgE autoantibodies aggravated disease in *FcγRIIB-/-, FcγRIIB-/- (x) Yaa* and MRL/Lpr mouse models of SLE	(16, 17, 32–34)
Th2 cells	- Frequency of Th2 cells were increased in *Ets1ΔCD4* mouse model of SLE - Th2 cells were detected in the kidneys of NZM2410 mouse model of SLE - IL-4 neutralizing antibodies administration or STAT6 deletion in NZM2410 mice resulted in the abrogation of nephritis symptoms - IL-4 transgenic B6C3F1 mice developed glomerulosclerosis associated with collagen deposition	(20, 26, 35–37)
Tfh2 cells	- IL-4 neutralization significantly decreased the frequencies of Tfh2 cells in *Ets1ΔCD4* mice while alleviating splenomegaly and reducing IgE autoanibody in serum	26
Extrafollicular helper T cells	- Involved in propagating autoantibody production in extrafollicular zone in MRL/Lpr mice	(38)
Anti-dsDNA IgE	- IgE autoantibodies were shown to be involved in aggravating disease in *Lyn-/-*, *FcγRIIB-/-*, *and FcγRIIB-/- (x) Yaa* mice	(7, 16)

### Interleukin-33

IL-33 is a nuclear factor expressed in the nuclei of structural cells and innate cells, which is released upon cell damage and necrosis (39). Once released, IL-33 propagates Th2 immune responses by activating ILC2s, Th2 cells, DCs, eosinophils, mast cells, and basophils. IL-33 also signals through structural cells, such as endothelial cells, epithelial cells, and fibroblasts (40). IL-33 signals through the heterodimeric complex consisting of interleukin-1 receptor-like 1 (IL1RL1 or ST2) and IL-1R accessory protein (IL-1RAcP). Due to its strong induction of type 2 responses, IL-33 is a central player in driving allergic disease (39). However, IL-33 is also involved in maintaining tissue homeostasis and tissue repair *via* activating ST2^+^ regulatory T (Treg) cells and ILC2s to produce amphiregulin (Areg) (41). In a different light, IL-33, in the presence of IL-12, can also indirectly promote type 1 responses by activating Th1, CD8 T cells, NK cells, and NKT cells to produce interferon-γ (IFN-γ) (39). Thus, IL-33 plays multiple roles in physiology and its function comes in various flavors in a context-dependent manner.

In SLE patients, serum levels of IL-33 proteins were elevated in SLE patients compared to healthy controls (42). However, serum IL-33 levels did not correlate with most clinical and laboratory characteristics of patients, including SLE disease activity index (SLEDAI) scores and anti-dsDNA autoantibody titers. In another study, Mok et al. found comparable levels of IL-33 protein in the serum of SLE patients and healthy controls (43). Thus, it seems the systemic release of IL-33 does not play a role during the pathogenesis of SLE. On the other hand, Georgakis et al. discovered an increase of neutrophil extracellular traps (NETs) decorated with bioactive IL-33 in the blood of SLE patients, which showed a positive correlation with SLEDAI scores (15). NETs decorated with IL-33 were potent in activating plasmacytoid dendritic cells (pDCs) to produce type 1 interferons (IFN-I), which play key roles during SLE pathogenesis (15, 44). Interestingly, this group also detected IL-33-decorated NETs in inflamed skin and inflamed kidney of SLE patients. However, the role played by IL-33-decorated NETs at these sites requires further investigation. Mouse studies mirror discoveries made in humans as treatment of IL-33 inhibitory antibodies alleviated lupus symptoms in MRL/Lpr mice (30). It will be interesting to evaluate whether the therapeutic effect of IL-33 neutralization is due to suppression of IL-33-decorated NETs in MRL/Lpr mice. There are, however, some discrepancies in animal models as early treatment of recombinant IL-33 to NZB/W F_1_ mice alleviated SLE symptoms (31). As explained earlier, the effect of IL-33 is highly context-dependent and it seems IL-33 is pathogenic in SLE only when in complex with NETs. Although the role of IL-33 decorated NETs for activating pDCs and inducing IFN-I has been identified, their roles in inducing type 2 immune responses in SLE requires further investigation.

### Basophil

Basophils are one of the rarest cell types in the body, making up less than 1% of the circulating white blood cell population. Due to their rarity, basophils were considered less important for the pathogenies of SLE. However, the role of basophils has been re-examined in the past few decades. The first evidence was provided by a study on Lyn-deficient mice (*Lyn* ^-/-^) mice. Lyn is a Src family protein tyrosine kinase and it is reported that *Lyn^-/-^* mice develop spontaneous autoimmunity and lupus nephritis in old age (16). This study further demonstrated that activated basophils and IgE autoantibodies play essential roles in amplifying autoantibody production and the development of lupus nephritis. Mechanistically, basophils were activated by autoreactive IgE and recruited to secondary lymphoid organs (SLO) where they promoted Th2 cell differentiation and the production of autoantibodies (16) (**Figure 1**). The role of basophil in aggravating SLE was further demonstrated in *FcγRIIB^-/-^*, *FcγRIIB^-/-^ (x) Yaa* and MRL/Lpr mouse models of SLE as well as in the pristine-induced model of SLE (17, 32–34). This phenomenon was also discovered in SLE patients where basophil counts were reduced in circulation while they accumulated in the lymph nodes and spleen of SLE patients (16, 17). The recruitment of basophils to SLO requires activation by factors such as autoreactive IgE and Prostaglandin D2 (PDG2) (16, 34). Upon activation, basophils upregulate CD62L, CCR7, and CXCR4 which leads to their recruitment to SLOs (17).

Once recruited into SLOs, basophils are involved in activating T cells, B cells, and pDCs. IL-4 is indispensable for the induction of Th2 cells, yet the initial source of IL-4 has remained elusive. Currently, basophils are considered the potential primary source of IL-4 *in vivo* (18). Thus, basophils are highly effective in polarizing Th2 cell differentiation while inhibiting differentiation into Th1 cells (45). Similarly, T follicular helper type 2 (Tfh2) cells also require IL-4 for its differentiation (26). Therefore, basophils might also play a central role during the induction of Tfh2 cells; however, the main cell types involved during the differentiation of Tfh2 cells are yet to be determined. Tfh2 cells are the main cell type that induces IgE class switching of B cells, therefore, the induction of Tfh2 cells is a key step for the induction of autoreactive IgE responses. This is discussed in more detail later. In addition to the production of Th2 related cytokines, basophils can also express IL-6 upon activation. IL-6 is required for driving Th17 differentiation, and indeed Pan et al. demonstrated basophils isolated from SLE patients but not from controls could induce the differentiation of Th17 cells (17).

In addition to activating T cells, basophils are also involved in activating B cells. Upon activation, basophils upregulate expression of molecules such as B-cell activating factor (BAFF), and A proliferation-inducing ligand (APRIL), thereby interacting with B cells in a contact-dependent manner (46). Moreover, IL-4 is a survival factor and differentiation factor for B cells. Thus, basophil derived IL-4 is important for the survival of B cells and the differentiation of B cells to plasma cells, and isotype switching of B cells to IgE isotype (47). Indeed, culture of basophils isolated from SLE patients with B cells induced the secretion of anti-nuclear IgG and anti-nuclear IgE, in the absence of T cells (17). Thus, basophils are involved in the differentiation and activation of both T cells and B cells in SLOs.

### T Helper 2 Cells

Th2 cells mainly mediate type 2 immune responses. Type 2 immune responses are critical in eradicating extracellular parasites but are also involved in diseases such as asthma, food allergy, and atopic dermatitis. IL-4, IL-5, and IL-13 are the main effector cytokines secreted by Th2 cells. IL-5 signals through the IL-5R, which is highly expressed in eosinophils and partly in basophils and mast cells. However, the function of IL-5 is quite specific for the biology of eosinophils and is involved in the activation, survival, and differentiation of eosinophils (48). IL-4 and IL-13 drive most of the hallmarks of Th2 responses such as IgE production, M2 macrophage differentiation, smooth muscle contractility, mucus production, and recruitment of innate cells to the site of inflammation (49). IL-4 and IL-13 play redundant roles as they are usually co-expressed by lymphocytes, and they share the IL-4 receptor alpha (IL-4Rα). In terms of IgE production, both IL-4 and IL-13 play important roles. Indeed, *Il4 ^-/-^* and *Il13 ^-/-^* mice show partial IgE reduction while *Il4^-/-^/Il13^-/-^* double knockout mice and *Il4r^-/-^* mice show the highest reduction in IgE levels (35).

Th2 cells produce a high amount of IL-4 and IL-13, therefore, they were considered important for the shaping of humoral immune responses (50). However, the concept of Th2 mediated-IgE production and B cell activation must be revisited since recent studies prove that Tfh2 cells are the main contributors to IgE induction (26, 51, 52). Nevertheless, Th2 cells and Tfh2 cells share many characteristics, such as the expression of GATA-3 and the requirement of IL-4 cytokine for its differentiation. Therefore, Th2 cells might aid the induction of Tfh2 cells in a paracrine manner by providing a steady amount of IL-4 in the T cell zone. Moreover, the frequency of Th2 cells is increased in SLE mouse models and SLE patients, implying that they may have a role in the pathogenesis of SLE, be it independent of B cell activation (16, 26). Indeed, the role of Th2 cells in end-organ damage is further discussed below.

### T Follicular Helper 2 Cells

One of the critical features of SLE is the generation of autoantibodies against nuclear components. They form immune complexes (ICs) and cause chronic systemic inflammatory autoimmune disease. Antigen-specific autoreactive antibody-producing B cells develop in germinal centers (GCs) in which B cells undergo somatic hypermutation, selection, and differentiation into antibody-producing plasma cells and long-lived memory B cells (53). T follicular helper (Tfh) cells play critical roles in mediating GC reactions, from GC formation to the induction of high-affinity antibody-producing plasma cells. Since patients with SLE have somatically mutated high-affinity autoantibodies in serum, Tfh cells are likely to be involved with the pathogenesis of SLE (54, 55). The number of Tfh and Th17 cells positively correlated with disease activity in SLE patients, suggesting these cells mediate pathogenic responses during SLE (21–23). A clinical trial partially confirmed this hypothesis as administration of low-dose recombinant human IL-2 (rhIL-2) ameliorated disease activity in patients by increasing the number of regulatory T cells (Tregs) and decreasing the number of Tfh and Th17 cells (24).

Blood Tfh cells can be further subtyped into Th1, Th2, and Th17 subsets according to the expression of CXCR3 and CCR6 (51). Among these subsets, Tfh2 cells are involved in the pathogenesis and etiology of SLE. The frequencies of Tfh2 cells are significantly higher in active lupus patients, and the number of Tfh2 cells positively correlates with SLEDAI scores in SLE patients (25, 26). Tfh2 cells express GATA-3 and secrete high amounts of IL-4 (26). IL-4 acts as both an inducing factor and an effector molecule of Tfh2 cells as IL-4 neutralization significantly decreased the frequencies of Tfh2 cells in SLE mice while alleviating splenomegaly and reducing IgE autoantibody titers in SLE mice (26). In addition to IL-4, interferon-α (IFN-α) is also described to enhance the generation of circulatory Tfh2 cells in a model of adenovirus infection (56). The primary function of Tfh2 cells is the induction of IgE. Among the three Tfh subsets, only Tfh2 cells could promote IgE induction *in vitro* (51). Additionally, Tfh2 cells have superior capabilities to induce IgG production from B cells compared to Tfh1 cells. In line with this finding, Le Coz et al. and our group have observed Tfh2 cell frequencies have a positive correlation with serum anti-dsDNA IgG and anti-dsDNA IgE titers (25, 26). Additionally, Tfh2 cells were highly activated in SLE patients compared to the control group, as they had higher IL-4, IL-5, and IL-13 expressions and were better at inducing plasma cell differentiation of B cells *in vitro* (26). The exact mechanism underlying activation of Tfh2 cells in SLE patients is unknown, and requires further investigation.

### Extrafollicular Helper T Cells

IgE^+^ B cells follow a unique path of maturation compared to IgG^+^ B cells. IgE^+^ B cells experience a short period in germinal centers (GC) and quickly exit GCs to complete their maturation process in the extrafollicular zone (57). Therefore, IgE^+^ plasma cells (PCs) come in two waves. An initial wave of IgE^+^ PCs directly differentiate in the extrafollicular zone and have B cell receptors that have not undergone somatic hypermutations (SHM). The second wave of IgE^+^ PCs arises from IgE^+^ germinal center B cells (GC B) and have B cell receptors that have undergone SHM (57). As anti-dsDNA IgE and autoreactive IgE require SHM, we can confer that autoreactive IgE arises from GC-derived PCs. The cell types involved in the extrafollicular B cell response are not well described; however, the role of extrafollicular helper T cells has been recently described in the context of lupus.

Extrafollicular helper T cells have been detected in the spleens of MRL/Lpr mice (38). These cell types were important for driving the formation of extrafollicular plasmablasts in MRL/Lpr mice *via* CD40L, Icos, and IL-21. Similar to their Tfh cell counterparts, extrafollicular helper T cells required Bcl6 for their differentiation. However, they express distinct markers from Tfh cells: CD4^+^PSGL-1^low^CXCR5^low^CXCR4^+^. Extrafollicular helper T cells are also found in human tonsils, and they are CD4^+^PSGL1^hi^PD-1^hi^CXCR5^hi^ cells (58). They exhibit a transcriptionally distinct phenotype from Tfh cells and promote memory B cells to produce immunoglobulins *via* CD40L, IL-10, and IL-21. It is interesting to note that extrafollicular T helper cells express CXCR4 and that basophils activated by PGD2 turn on the same chemokine receptor. Thus, basophils have a high possibility of localizing at the extrafollicular region. In addition, considering the importance of IL-4 for the induction of IgE in B cells, the expression of IL-4 in extrafollicular helper T cells must be addressed in the future.

## Effector Phase

### T Helper 2 Cells

Once Th2 cell differentiation is completed within secondary lymphoid organs, they are recruited to the site of inflammation to elicit effector functions. Th2 cells are detected in kidney biopsies of lupus nephritis patients as detected by immunohistochemistry and in the kidneys of the NZM2410 mouse model of SLE (19, 36). IL-4 levels correlated with hypercellularity in nephritis patients suggesting Th2 cells play an active role in disease progression. Similarly, administration of IL-4 neutralizing antibodies or deletion of STAT6 in NZM2410 mice resulted in the abrogation of nephritis symptoms (20). Of note, IL-4 seems to be involved in glomerulosclerosis *via* direct interaction with kidney cells (36, 59). In a different model of IL-4 transgenic B6C3F1 mice, mice developed glomerulosclerosis associated with collagen deposition (37).

Alternatively, Th2 cells might implement its effector functions by recruiting and maintaining eosinophils in the kidney *via* releasing IL-5 cytokine in the kidney microenvironment. IL-5 is indispensable for the recruitment and survival of eosinophils. Interestingly, urinary IL-5 and eosinophiluria were increased in patients with lupus nephritis, suggesting IL-5 mediated recruitment of eosinophils occurs in nephritis patients (28). Thus, Th2 cells might mitigate effector functions *via* two tracks by interacting with kidney cells to induce glomerulosclerosis or secreting IL-5 to recruit eosinophils to the kidney.

### Anti-dsDNA IgE

The role of autoreactive IgE in SLE patients has been discussed since the 1970s in line with their abilities to promote basophils (60). IgE is the least abundant immunoglobulin isotype in a healthy individual and is commonly known to trigger type I hypersensitivity. Elevated levels of IgE reflect type 2 immune response since type 2 cytokines, such as IL-4, induce IgE production (61). Among 117 US and 79 French patients with SLE, about 50~60% of the cohort were positive for autoreactive IgE. These antibody titers correlated with disease activity and active nephritis, suggesting IgE is involved during the pathogenesis of SLE (7). Indeed, anti-dsDNA IgE levels show comparable disease predictive ability as anti-dsDNA IgG, while the combination of both parameters enhances prediction (7). Moreover, anti-dsDNA IgG and anti-dsDNA IgE are each risk factors for SLE in an independent fashion, suggesting they do not share identical pathways (47).

In line with human studies, there is ample evidence from mice studies highlighting the pathogenic role of IgE autoantibodies in SLE. IgE autoantibodies were involved in aggravating disease in *Lyn^-/-^*, *FcγRIIB^-/-^*, and *FcγRIIB^-/-^/Yaa* mice (7, 16). Deficiency of IgE in SLE mouse models resulted in the decrease of autoantibodies and amelioration of organ pathology. Mechanistically, IgE autoantibodies were involved in the activation of basophils (which is discussed above) and the activation of pDCs (62). pDCs were found to express FcϵRI, which is the high-affinity IgE receptor. Thus, pDCs could uptake dsDNA-IgE complexes in a FcϵRI dependent fashion, and sensed DNA in a toll-like receptor 9 (TLR9) dependent manner. Upon DNA sensing pDCs secreted a high amount of IFN-I. This pathway was physiologically relevant as the IFN-α inducing capacity of SLE serum was reduced upon IgE neutralization, *in vitro* (62).

While the role of IgE autoantibodies in amplifying inflammation is well studied, the role of IgE autoantibodies in mediating end organ damage is still elusive. A study conducted with lupus patients revealed that 35% of lupus nephritis patients showed IgE deposition in the kidney, and these patients had a poorer prognosis. Moreover, among patients with IgE deposition, the occurrence rate of lupus nephritis increased by up to 71% (27). This strongly suggests that IgE has a pathogenic role in the kidney. IgE is different from other immunoglobulins, as they cannot activate complement pathways. Instead, they act through binding and crosslinking high-affinity IgE (FcϵRI) receptors expressed on the surface of mast cells, basophils, and to a lesser extent on eosinophils (63, 64). As IgE are deposited in inflamed kidneys of lupus mice and patients, they are suspected to mediate local immune reactions and inflammation. However, the exact role played by IgE in the kidney remains to be addressed.

### Eosinophil

Eosinophils are innate immune leukocytes stimulated by IL-5, granulocyte-macrophage colony-stimulating factor (GM-CSF), and IL-33. They are the effector arm in Th2 related immune responses such as parasite infection and in Th2 associated diseases such as asthma and allergic responses (65, 66). Eosinophils are distributed in the bone marrow, blood, spleen, thymus, gastrointestinal tract, and uterus during steady state (67). However, under pathogenic conditions, they infiltrate into inflamed tissues and contribute to organ destruction by mediating local cytotoxic actions through the secretion of granule proteins (68). A recent study with lupus nephritis patients discovered an increase in urinary eosinophils along with the detection of eosinophil cationic protein and IL-5. Eosinophils also correlated with renal function and SLE disease activity, and authors suggest using urine eosinophil levels as a biomarker for active lupus nephritis (28). Furthermore, patients with eosinophiluria and tissue eosinophil infiltration were significantly more likely to progress to end-stage kidney disease. However, the precise relation between eosinophiluria and lupus nephritis is poorly understood. Blood eosinophil levels are also comparable between SLE patients and healthy controls. Nevertheless, these are exciting observations that call for further research into understanding the role of eosinophils in mitigating glomerular damage (28, 29, 69).

## Conclusion

Over 50% of SLE patients display a type 2-associated immune response, as evidenced by the detection of IgE autoantibodies. These IgE autoantibodies were clinically meaningful as IgE neutralization with omalizumab treatment alleviated disease severity. This review illustrates the players involved during the induction and effector phase of type 2 immunity in the context of SLE pathology. The induction phase likely consists of the release of IL-33-decorated NETs from neutrophils. NETs decorated with IL-33 can activate multiple innate cells containing IL-33R, such as pDCs and basophils. Upon activation, basophils express CD62L and CCR7 and are recruited to SLOs. In SLOs, basophils aid in polarizing T cells towards Th2 cells and Tfh2 cells while also activating B cells. Tfh2 cells are indispensable for activating and producing IgE-producing plasma cells. The exact mechanism leading to the induction of autoreactive IgE plasma cells is unknown. However, it will most likely be a result of loss of quality control in germinal centers or in the extrafollicular zone. Once IgE autoantibodies are produced, they further activate pDCs and basophils, forming a positive feedback loop. Alternatively, circulating IgE autoantibodies are deposited in the kidney. At this site, IgE can interact with eosinophils to signal the release of cytotoxic molecules such as cationic proteins. Th2 cells also infiltrate into the interstitial region of the kidney, where they directly interact with kidney cells to induce fibrosis and glomerulosclerosis. The success of IgE targeting therapy in patients has sparked interest in understanding the role of Th2 related responses in SLE. There are many missing links in this field; however, additional research will draw a clearer map of the immune response in type-2 subtyped SLE patients and will pave the way for developing novel therapies for Th2 associated SLE.

## Author Contributions

HK, CK, and S-HI conceptualized the article, reviewed the literature, and wrote the manuscript. All authors contributed to the article and approved the submitted version.

## Funding

HK is grateful for financial support from Hyundai Motor Chung Mong-Koo Foundation. CK is grateful for financial support from the Sejong Science Fellowship from the National Research Foundation of Korea (Grant number: 2021R1C1C2014337).

## Conflict of Interest

S-HI is the CEO of the company ImmunoBiome Inc.

The remaining authors declare that the research was conducted in the absence of any commercial or financial relationships that could be construed as a potential conflict of interest.

## Publisher’s Note

All claims expressed in this article are solely those of the authors and do not necessarily represent those of their affiliated organizations, or those of the publisher, the editors and the reviewers. Any product that may be evaluated in this article, or claim that may be made by its manufacturer, is not guaranteed or endorsed by the publisher.
